# Neoandrographolide inhibits mature osteoclast differentiation to alleviate bone loss and treat osteoporosis

**DOI:** 10.3389/fphar.2025.1466057

**Published:** 2025-02-11

**Authors:** Kai Tang, Wei Deng, Zhiying Huang, Simin Chen, Zilin Zhu, Shukun Lin, Lubin Zhong, Quanxin Zheng, Wenhua Zhao, Zhida Zhang, Ling Mo

**Affiliations:** ^1^ Guangzhou University of Chinese Medicine, Guangzhou, Guangdong, China; ^2^ First Affiliated Hospital of Guangzhou University of Traditional Chinese Medicine, Guangzhou, Guangdong, China; ^3^ The Laboratory of Orthopaedics and Traumatology of Lingnan Medical Research Center, Guangzhou University of Chinese Medicine, Guangzhou, Guangdong, China; ^4^ The Second Clinical College of Guangzhou University of Chinese Medicine, Guangzhou, Guangdong, China; ^5^ The Eighth School of Clinical Medicine of Guangzhou University of Chinese Medicine, Foshan, Guangdong, China; ^6^ Department of Spine Surgery, The Second Affiliated Hospital of Guangzhou Medical University, Guangzhou, Guangdong, China; ^7^ Guangzhou Medical University, Guangzhou, Guangdong, China; ^8^ The Third Affiliated Hospital of Guangzhou University of Chinese Medicine, Guangdong Research Institute for Orthopedics and Traumatology of Chinese Medicine, Guangzhou, Guangdong, China

**Keywords:** neoandrographolide, osteoclastogenesis, MAPK, NF-κB, PI3K/AKT, PPARγ, GSK3β

## Abstract

**Background:**

Osteoporosis (OP), as the prevalent systemic metabolic bone disease worldwide, progresses insidiously and slowly. The clinical discomfort and complications associated with OP impose a significant burden on patients. Therefore, finding more effective treatments for OP remains an urgent challenge.

**Method:**

We first conducted *in vitro* experiments to determine whether Neoandrographolide (NEO) exhibits cytotoxic or proliferative effects on bone marrow macrophages (BMMs) and to explore the specific timeframe during which NEO exerts its inhibitory action on osteoclast (OC) differentiation. Through Reverse Transcription Polymerase Chain Reaction (RT-PCR) and Western blot analysis, we examined the relative expression levels of genes and proteins associated with OC differentiation like CTSK,c-Fos,MMP9,NFATc1, and verified the underlying mechanisms. Finally, we performed *in vivo* experiments to further investigate the inflammation.

**Results:**

NEO exhibits no significant cytotoxic effects on BMMs at concentrations less than or equal to 30 μM while exerting inhibitory effects on OC differentiation during its early and middle stages. RT-PCR and Western blot results reveal that NEO suppresses the expression of genes and proteins including CTSK,c-Fos,MMP9,NFATc1. Western blot findings also indicate that NEO inhibits the phosphorylation of ERK, P38, JNK, and P65 but does not reverse the degradation of IκB-α. Additionally, NEO affects the phosphorylation of proteins in the PI3K/AKT, GSK3β, and PPARγ signaling pathways, demonstrating that NEO can inhibit OC formation through multiple pathways and targets. *In vivo* experiments further validated the *in vitro* findings by constructing an OP model, showing that NEO can mitigate bone loss induced by OC differentiation.

**Conclusion:**

NEO has the potential to serve as a therapeutic agent for OP by targeting multiple sites and inhibiting the formation of mature OC through various signaling pathways.

## 1 Introduction

Bone, as a systemic tissue, stays in dynamic equilibrium, continuously remodeling to support and protect organs. However, when the balance between OB and OC is disrupted, it can lead to skeletal diseases like bone hyperplasia, calcification, and OP (Raut et al., 2019).

OP is a systemic metabolic bone disease characterized by reduced bone mass, lower bone mineral density, and deteriorated bone microarchitecture. This leads to fragility fractures, back pain, and kyphosis. Commonly seen in clinical settings, OP is diagnosed when the bone mineral density score is ≤ −2.5 and can be classified as either primary or secondary (Foessl et al., 2023). Globally, OP affects about 18.3% of the population, with rates around 23.1% in women and 11.7% in men. The highest prevalence occurs in Africa at approximately 39.4%. Both modifiable factors such as high BMI, smoking, alcohol use, and poor nutrition, and non-modifiable factors like gender, age, and ethnicity influence OP (Azeez, 2023). OP complications severely impact patients’ quality of life and longevity while increasing societal healthcare costs. Thus, improving prevention and treatment is crucial to reduce its occurrence and progression.

In clinical practice, there are several medications used to treat OP, including bisphosphonates, denosumab, and teriparatide. Bisphosphonates inhibit farnesyl diphosphate synthase, affecting intracellular signaling in OC. Denosumab binds to RANKL, preventing its interaction with RANK and inhibiting the formation and differentiation of mature OC. Teriparatide stimulates the kidneys to produce active vitamin D, increasing calcium absorption in the intestines and reducing bone calcium loss; it also promotes OB differentiation and bone formation (Munoz et al., 2020; Yong and Logan, 2021).

However, long-term use of these medications often leads to side effects that can undermine their therapeutic effectiveness. Prolonged bisphosphonate treatment can cause heart discomfort, arrhythmias, and complications such as jawbone fractures and periprosthetic hip fractures (Foessl et al., 2023; Serino et al., 2024; Alimy et al., 2024). Denosumab is unsuitable for long-term use because stopping it can rapidly decrease bone mass and raise the risk of multiple fragility fractures. Therefore, denosumab is often used alongside bisphosphonates to mitigate these adverse effects (Lamy et al., 2019; Solling et al., 2023). Teriparatide commonly causes nausea, vomiting, limb pain, headaches, dizziness, and temporary increases in blood calcium levels. Studies suggest that teriparatide may heighten the risk of osteosarcoma (Kim et al., 2021; Krege et al., 2022). Thus, developing clinical medications with fewer side effects and greater therapeutic benefits is essential.

With the progress of modern pharmacological research techniques and centuries of Chinese traditional medicine development, the use of Traditional Chinese Medicine in contemporary clinical practice has expanded, its mechanisms have become clearer, and it has gradually gained public acceptance. For instance, Wen-Shen-Tong-Luo-Zhi-Tong Decoction induces exosomes in adipocytes to negatively regulate SPRY2. This promotes osteogenic and adipogenic differentiation of bone marrow-derived mesenchymal stem cells (BMSCs), enhances trabecular bone parameters, and reduces fat accumulation (Wang et al., 2023). Xianling Gubao capsules and their key components inhibit OC bone resorption and promote OB differentiation. They also raise sex hormone levels, significantly enhancing bone metabolism and density (Tang et al., 2021; Wong et al., 2021). Erxian Decoction regulates lipid metabolism in adipose tissue and promotes OB differentiation and proliferation via the IGF1/PI3K/AKT pathway. It restores the bone-fat balance, improves bone microstructure, and mitigates bone loss (Ma Y. et al., 2023; Zhang et al., 2023). Epimedium, a traditional herbal medicine often used clinically, promotes OB proliferation and differentiation, enhances bone metabolism, and reduces inflammation. Furthermore, Epimedium extract stimulates angiogenesis, improving nutrient and oxygen transport to the bones. This increase in blood flow boosts bone mineral content and aids in bone regeneration (Qin et al., 2005; Xie et al., 2015).

Given the inherent limitations of current therapeutic strategies for OP, the development of novel treatment targets and pharmacological interventions holds significant clinical and scientific value. Existing therapies face considerable challenges in terms of long-term efficacy, safety profiles, and patient compliance, underscoring the urgent need to expand therapeutic options.

Neoandrographolide (NEO, CAS: 27215-14-1, SMILES: CC1(CCCC2(C1CCC(=C)C2CCC3=CCOC3=O)C)COC4C(C(C(C(O4)CO)O)O)O), a diterpenoid compound derived from *Andrographis paniculata*, exhibits numerous bioactive functions. Extracts of *A. paniculata* can modulate the P62-Keap1-Nrf2 axis, increasing the survival rate of PC12 cells. This modulation promotes Nrf2 protein expression while inhibiting Keap1 protein, thereby reducing aluminum-induced neurotoxicity and cognitive impairment (Ma J. et al., 2023). Studies using molecular docking have shown that NEO has strong anti-inflammatory properties. It inhibits both the NF-κB and Bax/Bcl-2 signaling pathways, reducing inflammation and apoptosis in myocardial cells simultaneously (Ma J. et al., 2023; Liu et al., 2021a). Derivatives of NEO have been demonstrated to regulate mitochondrial ROS levels in A549 tumor cells, increase caspase-3 protein expression, reduce inflammation, protect against skin damage, and prevent skin aging (Liu et al., 2021b; Mussard et al., 2020; Zhang et al., 2020). Andrograpanin, a hydrolytic product derived from NEO, can inhibit LPS-induced iNOS levels and the release of pro-inflammatory cytokines (TNF-α, IL-6) through P38 inhibition, thereby suppressing macrophage inflammatory responses (Liu et al., 2008).

Considering that NEO has anti-inflammatory properties and regulates ROS levels, and considering the link between inflammation and OP—where chronic inflammation can accelerate bone loss—certain immune cells may release inflammatory cytokines that interact with OC via paracrine mechanisms to regulate their activity (Fischer and Haffner-Luntzer, 2022; Montalcini et al., 2013). This study aims to comprehensively investigate the mechanisms by which NEO inhibits mature OC formation and mitigates bone loss through multiple signaling pathways. By employing modern pharmacological approaches, *in vitro* and *in vivo* models, we seek to elucidate the intricate molecular processes underlying NEO’s potential therapeutic effects on OP.

## 2 Materials and methods

### 2.1 Materials

Neoandrographolide (CAS: 27215-14-1) was purchased from Chamface Biotech Biotechnology Co., Ltd. (Wuhan, China). Minimum Essential Medium α (MEM-α), Penicillin-Streptomycin Solution (P/S), and Fetal Bovine Serum (FBS) were from Thermo Fisher Scientific (Shanghai, China). Recombinant Mouse TRANCE/RANKL and Recombinant Mouse M-CSF were sourced from R&D Systems. SYBR Green Premix and Evo M-MLV Mix were from Accurate Biology. CaMK2 alpha, phospho-CaMK2 alpha, CAMK4, phospho-CaMK4, Calmodulin 1/2/3, phospho-Calmodulin 1/2/3 were from the Bioss (Beijing, China); c-Fos,MMP9, CTSK, NFATc1,p38, Phospho-p38, ERK1/2, Phospho-ERK1/2, JNK1/2/3, Phospho-JNK1/2/3, P65, Phospho-p65, PI3K, Phospho-PI3K, AKT, Phospho-AKT, GSK3β, Phospho-GSK3β, PPARγ, Phospho-PPARγ,β-actin were all purchased from Affinity Biosciences Biotechnology (Jiang Su, China). DAPI, CCK-8, and Tartrate-Resistant Acid Phosphatase (TRAP) Stain Kits were sourced from Solarbio. (Beijing, China). CAMK4, Phospho-CAMK4, CAMK2, and Phospho-CAMK2 were purchased from Beijing Bioss Biotechnology Co., Ltd. (Beijing, China).

### 2.2 Extraction of murine bone marrow macrophages (BMMs)

Six-to eight-week-old C57BL/6J mice (provided by the Animal Experiment Center of Guangzhou University of Chinese Medicine, Ethics Number: 20,240,524,004) were euthanized using CO₂ inhalation followed by cervical dislocation under sterile conditions. Both lower limbs were collected, and excess muscle tissue was removed. The bone marrow was flushed out with 2.5 mL syringe, and after pipette homogenization, the suspension was centrifuged at room temperature at 1,000 rpm for 5 min. The pellet was resuspended in α-MEM medium (containing 10% FBS, 1% P/S, and 50% M-CSF) and transferred to 100 mm culture dish. Medium changes were performed every other day until the cells reached 85%–90% confluence. Subsequently, the cells were trypsinized, counted, plated, and prepared for further operations.

### 2.3 Cytotoxicity/proliferation

Seed mature BMMs in a 96-well plate at a density of 1 × 10^4^ per well. On the following day, add different concentrations of NEO (30 μM, 20 μM, 10 μM, 5 μM, 2.5 μM, 0 μM) and incubate for 48 h. Afterward, add 10 μL of CCK-8 solution to each well and incubate it in the dark for another 2 h. Measure the absorbance of each group at 490 nm using a microplate reader.

### 2.4 TRAP staining

Seed BMMs at a density of 5 × 10^3^ per well in a 96-well plate. On the second day, add culture medium containing 50 ng/mL M-CSF and 50 ng/mL RANKL, along with different concentrations of NEO (30 μM, 20 μM, 10 μM, 5 μM, 0 μM) to induce differentiation of BMMs into mature OC. Replace with fresh medium every other day until mature OC formation is observed around day six. Fix cells with 4% paraformaldehyde at room temperature and then perform TRAcP staining. Photograph using a fluorescent inverted microscope; consider cells with ≥3 nuclei as mature OC.

Seed BMMs into a 96-well plate and allow them to adhere. On days one, three, and five of differentiation into mature OC, add culture medium containing 50 ng/mL M-CSF and 50 ng/mL RANKL with different concentrations of NEO (30 μM or 0 μM) until OC maturation is complete. Then fix the cells, and photograph them for record-keeping and statistical analysis.

### 2.5 F-actin staining

Cells were seeded into 96-well plates. The next day, medium containing 50 ng/mL M-CSF, 50 ng/mL RANKL, and varying concentrations of NEO (30 μM, 15 μM, and 0 μM) was added. Once mature OC formed, cells were fixed with 4% paraformaldehyde for 10 min. Membranes were permeabilized with 100 μL of 0.5% Triton X-100 per well followed by blocking with 5% BSA for another 10 min at room temperature. In light-protected conditions, F-actin staining was applied and incubated overnight at 4°C. The following day, nuclei were stained with DAPI. The fluorescence intensity of each group was observed and photographed using the Nikon confocal microscope. F-actin ring areas and OC numbers in each group were quantified.

### 2.6 Reverse transcription polymerase chain reaction

BMMs were seeded at a density of 1 × 10⁵ per well in 6-well plates. The next day, different concentrations of NEO (30 μM, 15 μM, 0 μM) were added to induce BMM differentiation into mature OC. After about 6 days, when mature OC had formed, each well received 1 mL Trizol lysis reagent. Following a 30-minute incubation, we added 200 µL chloroform and centrifuged at 12,000 rpm and 4°C for 15 min. The RNA was reverse-transcribed into cDNA using Evo M-MLV and amplified with SYBR Green via RT-PCR. Quantitative analysis was conducted using the 2^-△△Ct^ method. The primer sequences for each gene are as follows (Deng et al., 2023; Liu et al., 2019): NFATc1 (5′-GGA​GAG​TCC​GAG​AAT​CGA​GAT-3'; 5′-TTG​CAG​CTA​GGA​AGT​ACG​TCT-3′); c-Fos: (Forward: 5′-GCG​AGC​AAC​TGA​GAA​GAC-3'; Reverse:5′-TTGAAACCCGAGAACATC-3′); CTSK (Forward: 5′-GGGAGAAAAACCTGA AGC-3'; Reverse: 5′-ATTCTGGGGACTCAGAGC-3′),MMP9 (Forward: 5′-CGTGTCTG GAGATTCGACTTGA-3′; Reverse: 5′-TTG​GAA​ACT​CAC​ACG​CCA​GA-3′).

### 2.7 Long-term Western blot

BMMs were seeded into 6-well plates. After cell adhesion the next day, medium containing 50 ng/mL RANKL and varying concentrations of NEO (30 μM, 0 μM) was added on days 1, 3, and 5 to induce OC differentiation. The medium was replenished every other day until mature OC formed in approximately 6 days. Then, 200 μL of lysis buffer was added to each well for protein extraction and quantified using BCA assay kit. Proteins were separated via SDS-PAGE, transferred onto PVDF membranes, and blocked with 5% BSA. Membranes were incubated with primary antibodies overnight at 4°C. The following day, secondary antibodies were incubated at room temperature; protein bands were then visualized using the ECL method and quantified with ImageJ software.

### 2.8 Short-term Western blot

BMMs were seeded into 6-well plates until they reached about 90% confluence. NEO (30 μM) was added at intervals of 0, 10, 20, 30, and 60 min. After NEO stimulation, proteins were extracted and incubated with primary antibodies as previously described for quantitative analysis.

### 2.9 Animal experiments

Female SPF-grade C57BL/6J mice, aged around 6–8 weeks, from the Animal Experiment Center of Guangzhou University of Chinese Medicine (the Ethics Number: 20,240,524,004) were randomly divided into sham surgery group, OP group, and drug stimulation group (low concentration and high concentration). The sham surgery group had adipose tissue near the ovaries removed; the OP group had bilateral ovariectomy to establish an OP model (Idris, 2012). After surgery, they were observed for 1 week to ensure no abnormal symptoms appeared. The drug stimulation groups were subjected to drug intervention according to low concentration (15 mg/kg) and high concentration (30 mg/kg) standards. Approximately 8 weeks later, the mice were overdosed with 0.3% sodium pentobarbital by intraperitoneal injection and then euthanized by cervical dislocation. The lower limbs were fixed in 4% paraformaldehyde, followed sequentially by Micro-CT scan analysis and immunohistochemical staining based on previous literature references (Liu et al., 2019; Bouxsein et al., 2010). Quantitative analysis indicators include BV/TV (Bone volume/Total volume), Tb. N (Trabecular number), Tb. The (Trabecular thickness), and Tb. Sp (Trabecular separation).

## 3 Statistical analysis

The results are presented as mean ± standard deviation. Statistical analyses were performed using SPSS 24.0 software; comparisons between two independent groups were made using unpaired *t*-tests, while comparisons amongst multiple independent groups employed the appropriate one-way or two-way ANOVA; a p-value of <0.05 was considered statistically significant and denoted by “*”. Data visualization was carried out using GraphPad Prism7 software.

## 4 Result

### 4.1 The CCK-8 result

**FIGURE 1:**
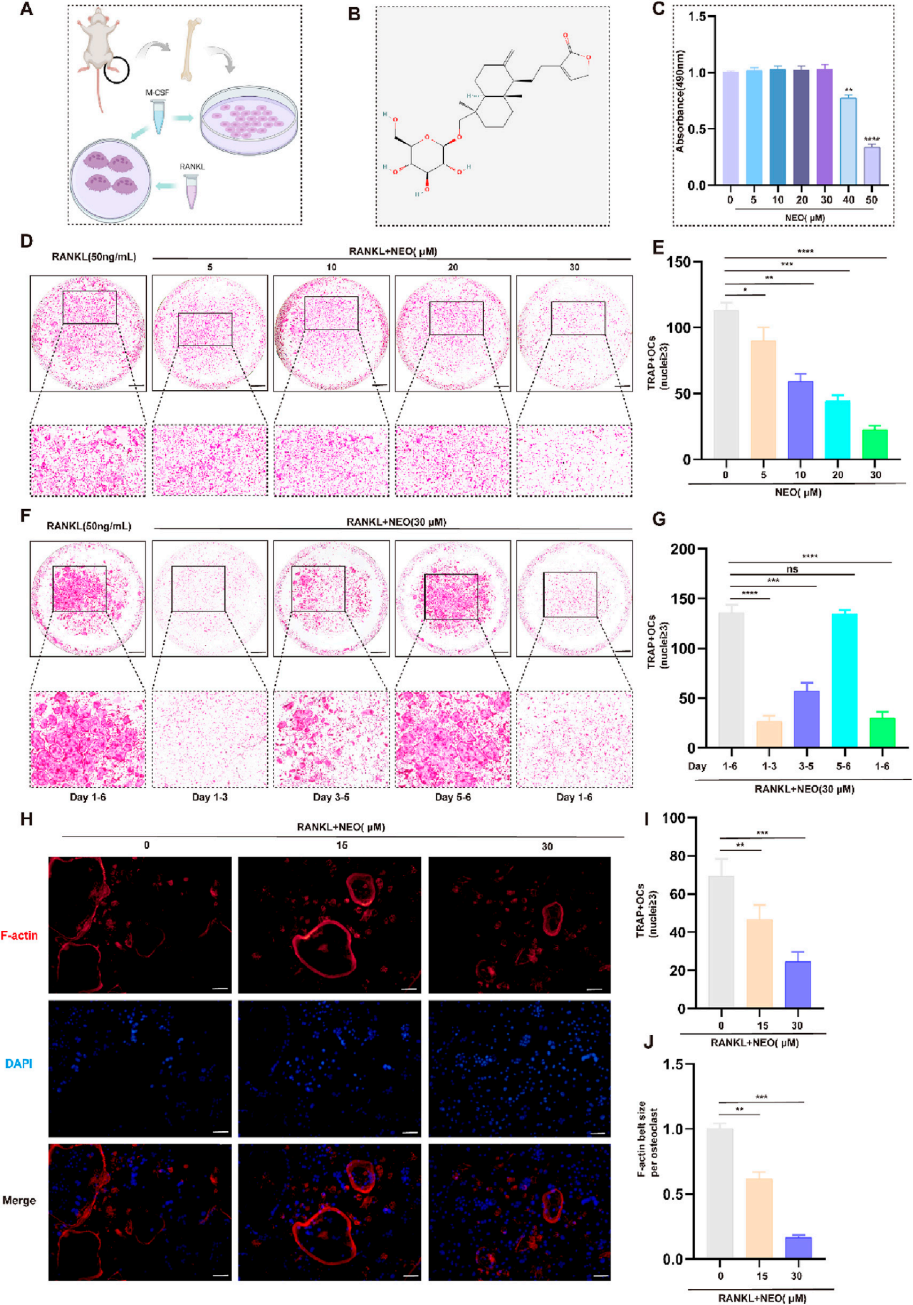
NEO inhibits OC formation and differentiation. **(A)** Schematic diagram of BMM cell incubation; **(B)** Chemical structure formula of NEO; **(C)** CCK-8 assay results; **(D, E)** NEO can inhibit the formation of mature OC, with the inhibitory effect showing a positive correlation with increasing concentration; **(F, G)** During the process of mature OC formation, the inhibitory effect of NEO is more pronounced in the early and middle stages but not significant in the later stage; **(H–J)** NEO can inhibit F-actin ring formation and nuclear aggregation.*:*p* < 0.05, **:*p* < 0.01, ***:*p* < 0.001 compared to the RANKL group. (n = 3). NEO, Neoandrographolide. Scale Bar = 200 μm.

Using a 48-hour CCK-8 assay to detect whether NEO has cytotoxic effects on BMM and its safe concentration. Figures 1A, B respectively represent the extraction and incubation schematic of BMM and the chemical molecular structure of NEO. As depicted in Figure 1C, NEO demonstrates no significant proliferative or cytotoxic effects on BMMs at concentrations up to and including 30 μM. However, a pronounced cytotoxic trend becomes evident at concentrations of 40 μM and 50 μM. Consequently, 30 μM was designated as the maximum effective concentration for subsequent experiments. Figures 1D, E use TRAP staining experiments to detect whether NEO inhibits RANKL-induced mature OC differentiation, as well as the inhibition trend. The results show that NEO can significantly inhibit the formation trend of OC; the higher the concentration, the stronger the inhibitory effect.

The osteoclastogenesis process typically extends over a 6-day period. To investigate the stage-specific effects of NEO, BMMs were exposed to RANKL(50 ng/mL) and NEO (30 μM) at distinct temporal intervals (days 1-3, 3-5, and 5-6) during the differentiation process. As illustrated in Figures 1F, G, significant suppression of mature OC formation was observed during both the early phase (days 1-3) and intermediate phase (days 3-5), suggesting that NEO’s inhibitory action on osteoclastogenesis is primarily manifested during these initial and intermediate stages of differentiation. Besides, analysis of F-actin staining corroborated NEO's inhibitory effects on cytoskeletal organization and nuclear clustering in mature OC, demonstrating concordance with the observations obtained from TRAP staining assays (Figures 1H–J).

### 4.2 NEO inhibits the expression levels of genes and proteins related to OC differentiation

**FIGURE 2:**
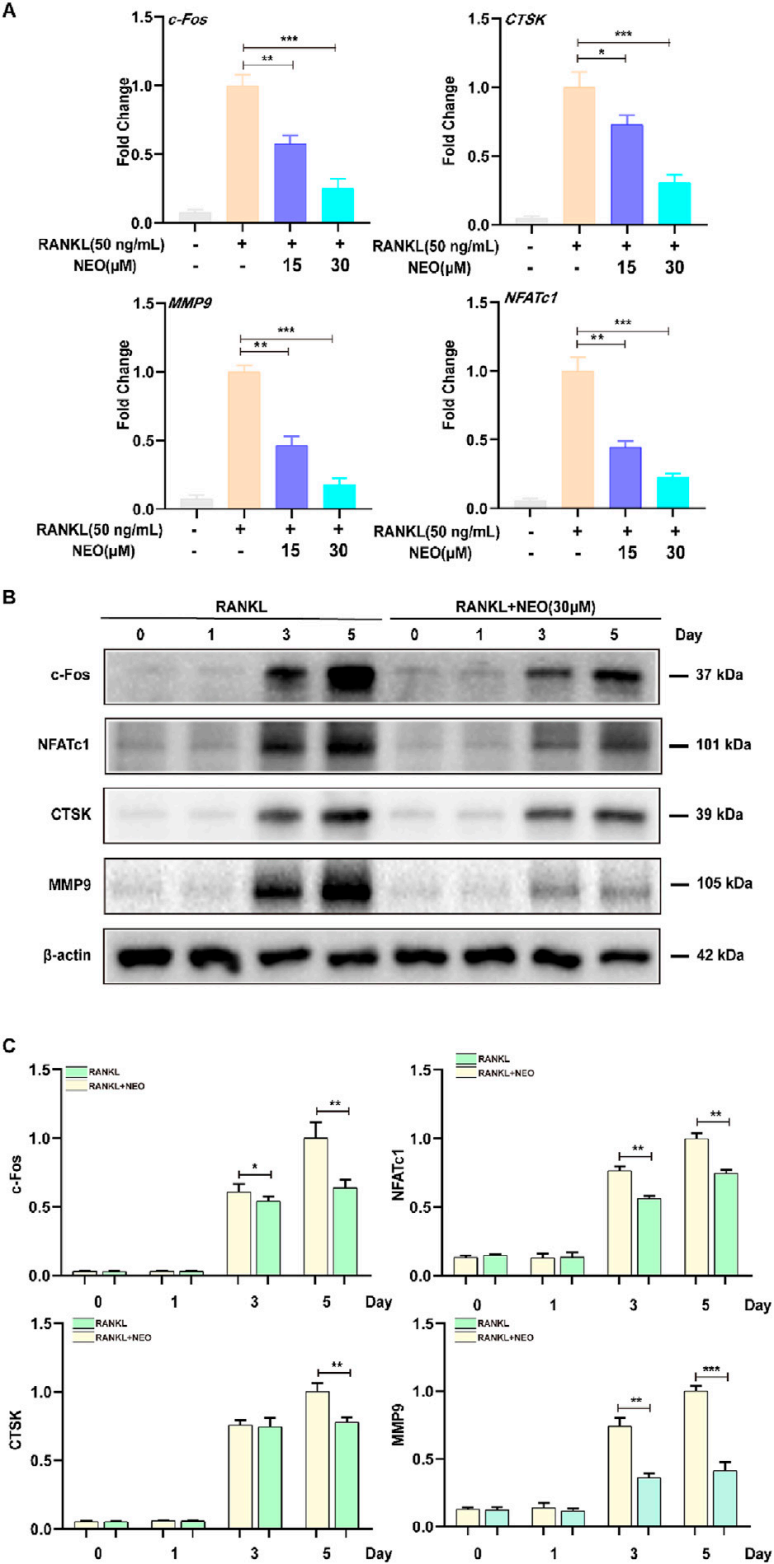
NEO illustrates the expression of genes and proteins related to the inhibition of OC formation. **(A)** NEO can inhibit the relative expression levels of the genes c-Fos, CTSK, MMP9, and NFATc1; the higher the concentration, the more pronounced the inhibitory effect. **(B, C)** NEO exhibits varying effects on the proteins c-Fos, NFATc1, CTSK and MMP9 at different time points.*:*p* < 0.05, **:*p* < 0.01, ***:*p* < 0.001 compared to the RANKL group. NEO, Neoandrographolide. (n = 3).

During OC differentiation, various transcription factors play important roles in OC formation, including NFATc1, CTSK, C-FOS, MMP9, etc. We used RT-PCR and Western blot experiments to detect the effects of NEO on related genes and proteins. As shown in Figure 2A, NEO inhibits the expression of related genes in a concentration-dependent manner; the higher the concentration, the more significant the inhibitory effect. Figures 2B, C show that NEO inhibits the relative expression levels of c-Fos,MMP9 and NFATc1 on days 3 and 5, while for CTSK it only shows an inhibitory effect starting from day 5.

### 4.3 NEO inhibits MAPK protein phosphorylation expression

**FIGURE 3:**
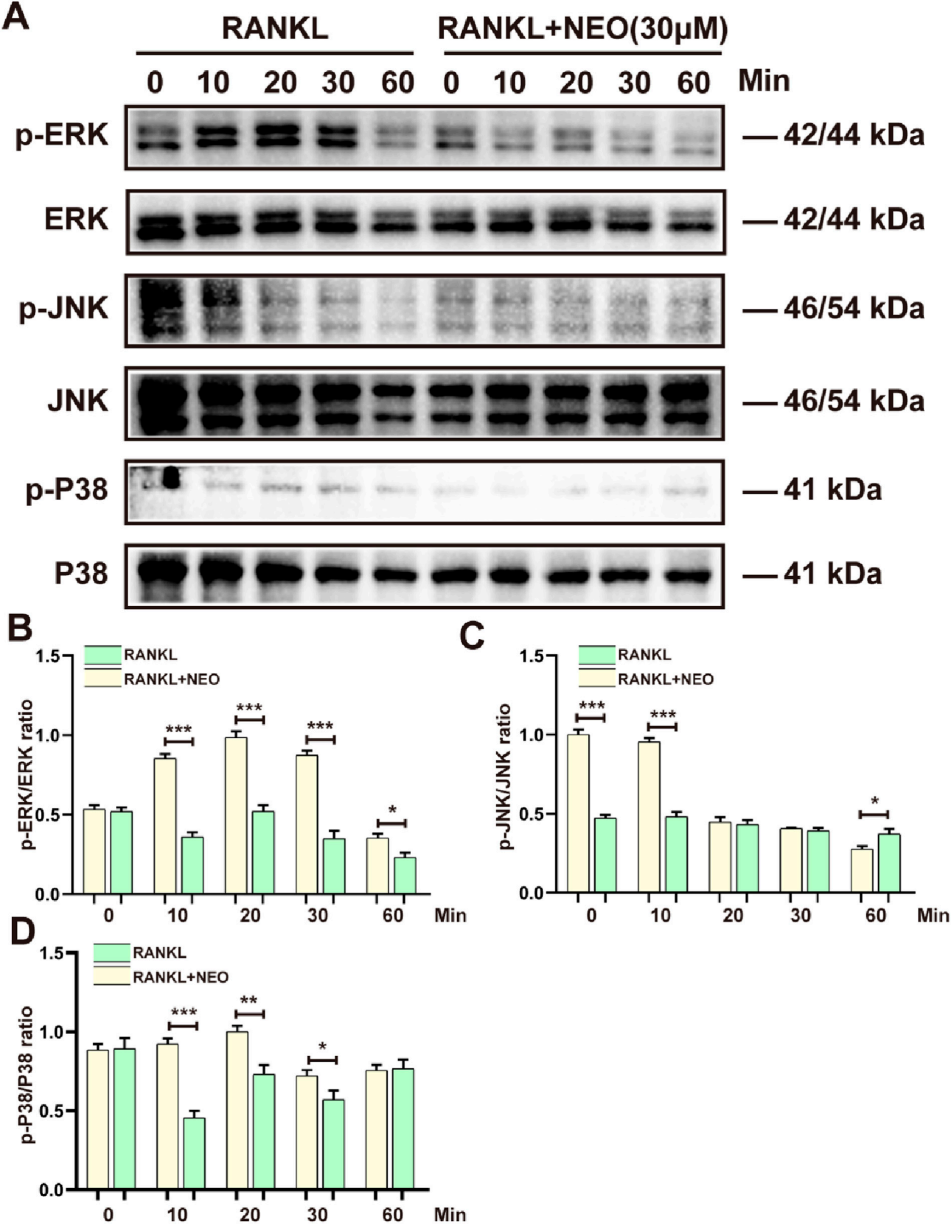
NEO inhibits the expression of MAPK signaling pathway proteins. **(A)** P38, ERK, and JNK show varying degrees of inhibition under NEO stimulation; **(B–D)** Statistical results related to the grayscale values of relevant proteins.*:*p* < 0.05, **:*p* < 0.01, ***:*p* < 0.001 compared to the RANKL group. NEO, Neoandrographolide. (n = 3).

To further explore the mechanism by which NEO inhibits OC differentiation, we examined the expression levels of the MAPK signaling pathway under NEO treatment. As shown in Figure 3A, NEO inhibited the phosphorylation levels of ERK and P38 at 10 min, 20 min, and 30 min. Additionally, at 60 min, NEO also inhibited p-P38 expression. Regarding p-JNK, NEO significantly inhibited its expression level at 10 min; however, this inhibitory effect disappeared by 60 min. At that time point (60 min), under stimulation with NEO, the expression level of p-JNK showed an upward trend. Figures 3B–D present bar chart analysis results for related proteins.

### 4.4 NEO has an inhibitory effect on the NF-κB/PI3K-AKT signaling pathway

**FIGURE 4:**
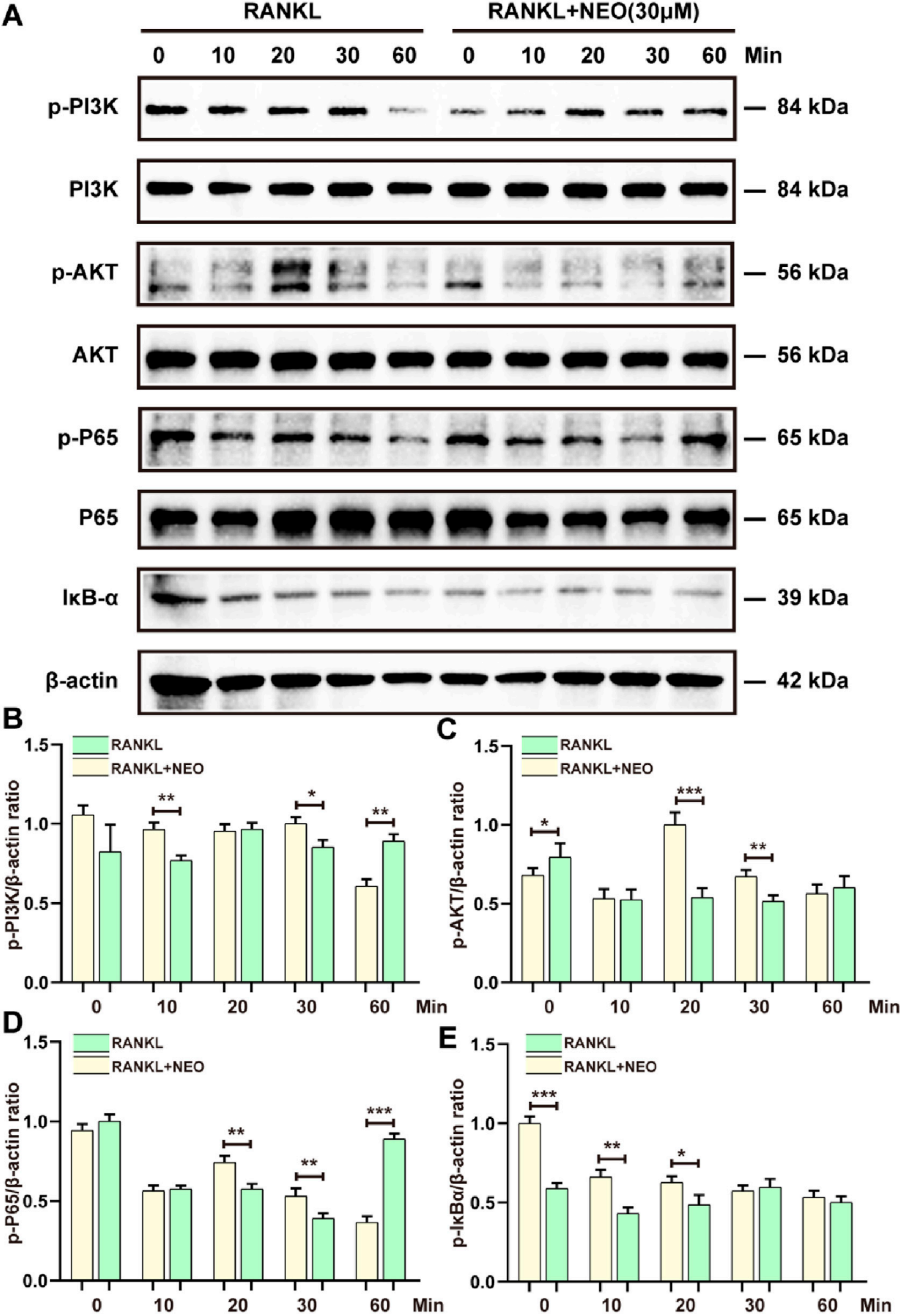
NEO regulates the NF-κB/PI3K-AKT signaling pathway. **(A)** NEO can affect the relative expression levels of PI3K, AKT, P65, and IκB-α proteins to varying degrees; **(B–E)** Analysis results of the grayscale values of related proteins.*:*p* < 0.05, **:*p* < 0.01, ***:*p* < 0.001 compared to the RANKL group. NEO, Neoandrographolide. (n = 3).

NF-κB/PI3K-AKT plays an important regulatory role in cell differentiation and proliferation. We examined the regulatory effects of NEO on the expression levels of proteins in these two signaling pathways. As shown in Figure 4A, NEO had an inhibitory effect on p-PI3K expression levels at the 10-min and 30-minute marks, but by the 60-minute mark, p-PI3K expression showed an upward trend, indicating that NEO does not have an inhibitory effect on p-PI3K expression at the 60-min mark. This is consistent with a similar inhibition trend observed for P65. NEO inhibited phosphorylated P65 expression at the 20-minute and 30-minute marks, but this inhibitory effect disappeared by the 60-minute mark. Regarding inhibition of p-AKT, it was most evident at the 20-minute and 30-minute marks; however, there was no significant effect observed at the initial time (0 min). Concerning IκB-α degradation phenomena, Western blot results indicated that NEO could not prevent RANKL-induced degradation of IκB-α. Figures 4B–E shows the statistical bar chart of the expression levels of related proteins.

### 4.5 NEO inhibits GSK3β/PPARγ protein expression levels

**FIGURE 5:**
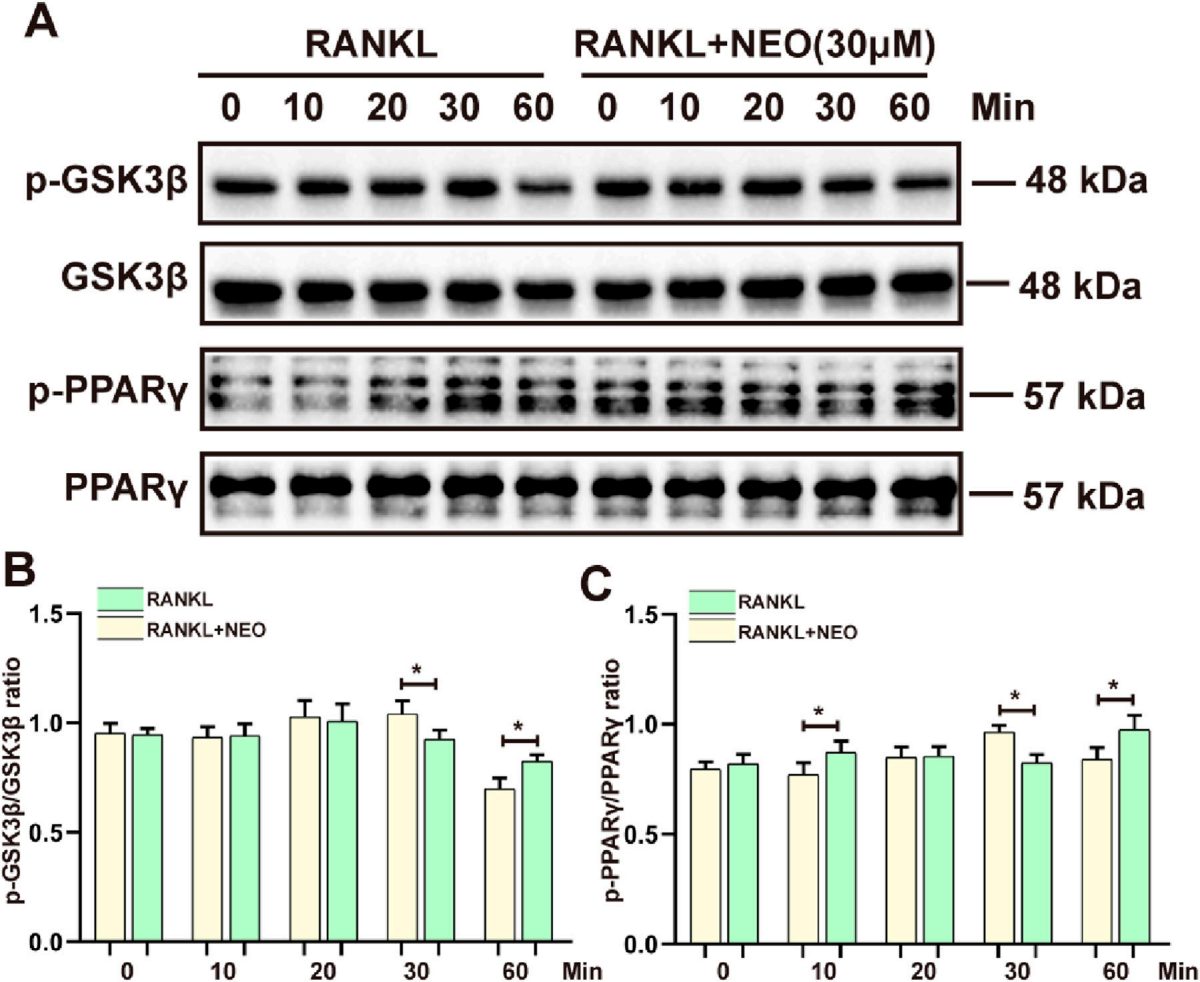
NEO inhibits the phosphorylation expression of GSK3β/PPARγ. **(A)** At different time points, NEO exhibits distinct effects on GSK3β/PPARγ; **(B, C)** Bar graphs for the quantitative analysis of the two proteins. *:*p* < 0.05, **:*p* < 0.01, ***:*p* < 0.001 RANKL to the control group. NEO, Neoandrographolide. (n = 3).

GSK3β and PPARγ, as two major cytokines, play a crucial role in various cell biological activities and energy metabolism related to cell fate (Fan et al., 2015; Wan, 2010). We also explored whether NEO would have an inhibitory effect on GSK3β/PPARγ. As shown in Figure 5A, the inhibitory effect of NEO on GSK3β is mainly concentrated at the 30-minute mark, while this inhibitory effect disappears at the 60-minute mark; For PPARγ, the inhibitory effect of NEO is also observed at the 30-minute mark but does not show significant inhibition effects at the 10-minute and 60-minute marks. Figures 5B, C display relevant statistical results of protein grayscale values.

### 4.6 NEO can activate calcium-related pathway proteins

**FIGURE 6:**
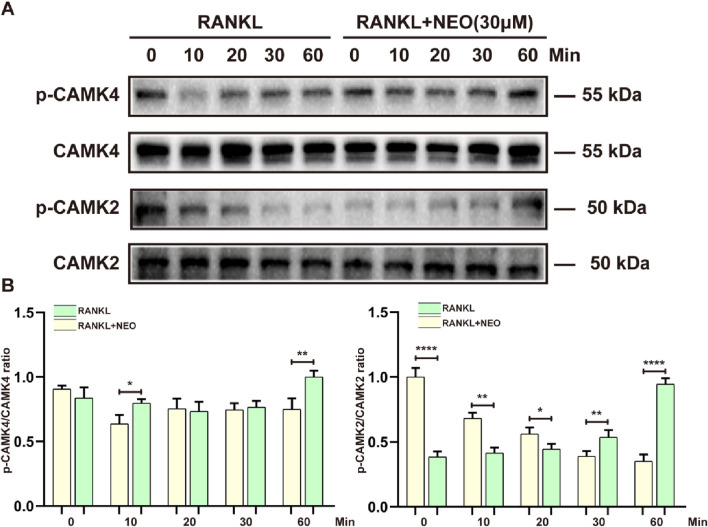
NEO regulates the expression levels of calcium-related proteins. **(A)** At different time points, NEO can have varying promoting or inhibiting effects on CAMK4 and CAMK2; **(B)** Bar chart of quantitative analysis of CAMK4 and CAMK2 proteins. *:*p* < 0.05, **:*p* < 0.01, ***:*p* < 0.001 compared to the RANKL group. NEO, Neoandrographolide. (n = 3).

During OC differentiation and involvement in cellular metabolism, related cell membrane channels such as calcium also play a key role, including CAMK4 and CMK2. As shown in Figures 6A, B, the effect of NEO on CMK4 is mainly observed at the 10th minute and the 60th minute. After NEO stimulation, the expression of CMK4 shows an upward trend. For CAMK2, NEO exhibits a significant inhibitory trend at 0 min, 10 min, 20 min, and 30 min; however, at the 60th minute, the expression of CAMK2 shows a clear upward trend. This indicates that NEO does not have an inhibitory effect on CAMK2 at the 60th minute.

### 4.7 NEO alleviates bone loss

**FIGURE 7:**
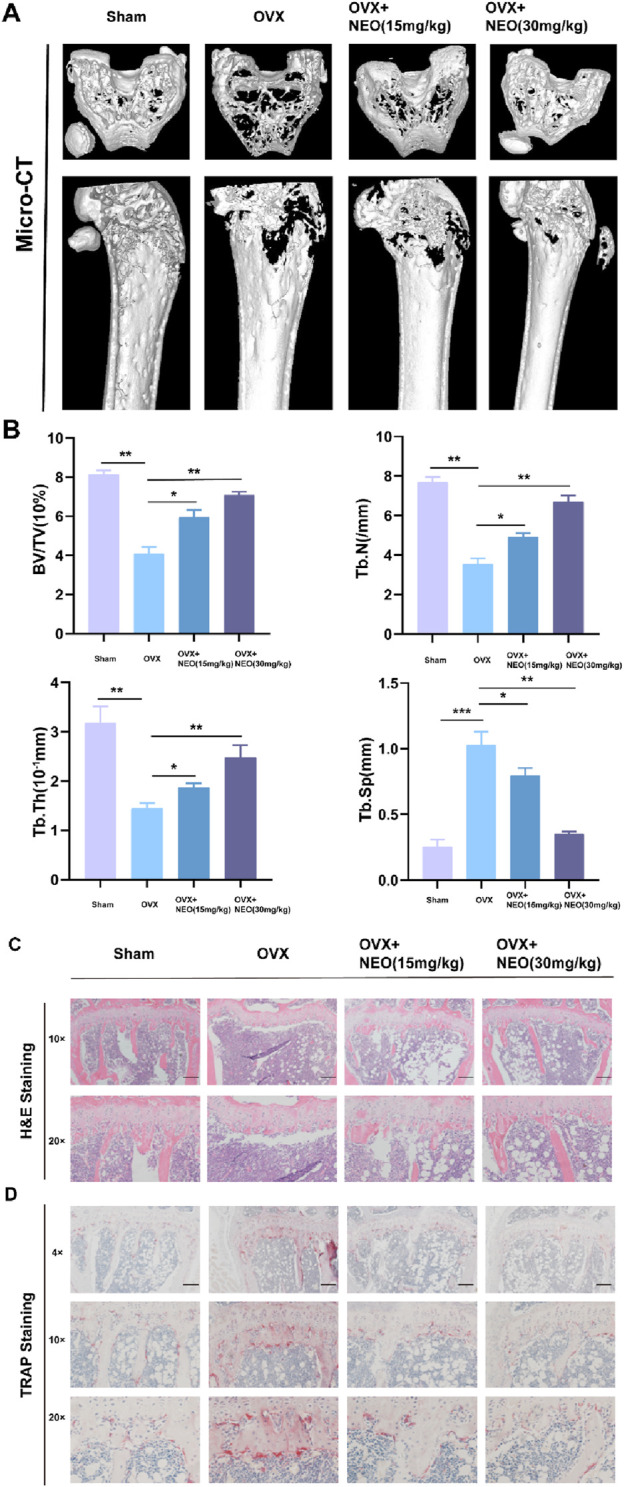
NEO can reduce bone loss and improve related parameters of trabecular bone. **(A, B)** Different concentrations of NEO have varying degrees of alleviating effects on bone loss and can also improve related parameters of trabecular bone. **(C, D)** HE and TRAP staining experiments indicate that NEO can inhibit the formation of mature OC. NEO, Neoandrographolide. (N = 8).

We investigated the mechanism of NEO in inhibiting OC differentiation through *in vitro* experiments and conducted further relevant validation through *in vivo* experiments. As shown in Figures 7A, B, compared to the Sham group, the bone mass of the OVX group showed a significant downward trend, but under NEO stimulation, bone loss was alleviated. HE and TRAP staining also demonstrated that NEO could significantly inhibit OC differentiation and improve trabecular parameters. This further confirmed that NEO could alleviate bone loss by inhibiting mature OC formation (Figures 7C, D).

## 5 Discussion

An excessive number of OC formations is a primary cause of OP. Numerous therapies, including drugs like denosumab and teriparatide, have been used to prevent and reduce bone resorption. However, the side effects of these medications can limit their effectiveness. Meanwhile, ongoing pharmacological research into natural substances has led clinicians to explore plant-based components for treating conditions caused by excessive bone resorption (Cai et al., 2024; Ramesh et al., 2021).

The differentiation of mature OC is concurrently regulated by various factors during their formation, which induce cell proliferation, survival, and differentiation, such as NFATc, CTSK, c-Fos, and MMP9 (Zhao et al., 2007). Under the stimulation of RANKL, the c-Fos signaling pathway mediated by the downstream MAPK/NF-κB facilitates the activation and nuclear expression of NFATc, thereby inducing the maturation of OC (Aliprantis et al., 2008). NFATc is simultaneously influenced by the calcium ion signaling pathway. The phosphorylation of calmodulin promotes self-amplification of the NFATc1 gene, which induces transcription of downstream genes CTSK and Acp5. However, inhibiting NFATc1 activity significantly reduces the number of mature OC, leading to an abnormal increase in bone mass and increased bone rigidity (Asagiri et al., 2005; Omata et al., 2023).

In this study, the combined results of *in vitro* and *in vivo* experiments demonstrate that NEO can inhibit the formation and differentiation of mature OC. It can also suppress OC activity through the MAPK/NF-κB/PI3K-AKT/PPARγ-GSK3β signaling pathways stimulated by RANKL, as well as the relative expression levels of NFATc1, c-Fos, CTSK, and MMP9 genes and proteins, thereby alleviating bone loss and degradation.

The MAPK signaling cascade is crucial for cell proliferation, survival, and differentiation. The combined action of M-CSF and RANKL triggers ERK phosphorylation, which enhances bone resorption and promotes the fusion and migration of OC precursors (Fujii et al., 2022); When M-CSF binds to its receptor c-Fms, it induces c-Fms phosphorylation. This phosphorylated receptor then interacts with growth factor receptor-binding protein-2 (Grb2), leading to ERK activation and ultimately regulating OC maturation (He et al., 2011); ERK also plays the key role in promoting OC differentiation under the influence of inflammatory cytokines such as IL-1β, IL-6, and IL-1α (Teitelbaum and Ross, 2003; Lee et al., 2018).

JNK mainly regulates OC apoptosis. Inhibiting JNK effectively blocks the RANKL/RANK/TRAF6 signaling pathway. When the JNK/C-Jun axis is overly silenced, BMMs cannot fully differentiate into mature OC even with continuous RANKL exposure, indicating that JNK is crucial for transforming mononuclear OC precursors into multinucleated mature OC (An et al., 2019; Dong et al., 2022). P38 is crucial in bone remodeling. When P38 was knocked out in mice, bone mass abnormally increased, OC numbers dropped, and bone resorption activity decreased (Cong et al., 2017). The study results show that NEO significantly inhibits p-ERK phosphorylation at 10, 20, 30, and 60 min. It also reduces JNK phosphorylation at 10 and 60 min while simultaneously inhibiting the relative expression of p-P38. These findings align with previous research indicating that external stimuli can suppress RANKL-induced P38 activation (Lee et al., 2018).

The NF-κB signaling pathway is closely associated with the mechanisms of inflammatory and osteolytic diseases and plays a critical role in OP treatment. This connection is evident during RANKL-induced OC differentiation, where P65 modulates JNK apoptosis to promote OC maturation (Abu-Amer et al., 2001). When RANKL activates the IκB kinase (IKK) complex, it triggers IκBα phosphorylation, leading to its ubiquitination and proteasomal degradation. These actions enable the nuclear expression of RelA-containing dimers, enhancing the transcription of OC target genes (Abbas and Abu-Amer, 2003; Vaira et al., 2008). Our study shows that NEO can inhibit P65 phosphorylation at 20 and 30 min, but this effect fades by 60 min. Additionally, NEO does not prevent IκBα degradation.

The PI3K-AKT signaling pathway can promote OC differentiation. This study shows that NEO inhibits PI3K/AKT phosphorylation at 10, 20, and 30 min. However, by the 60th minute, its inhibitory effect on PI3K phosphorylation is no longer observed. Mediated by RANKL/M-CSF, this pathway activates in OC precursor cells, guiding monocytes to mature into multinucleated cells (Jin et al., 2019; Leibbrandt and Penninger, 2008). Substantial evidence supports the inhibitory effects of PI3K-AKT inhibitors on both OC and OB. Additionally, factors secreted by OC can enhance MC3T3 cell differentiation via the PI3K/AKT signaling pathway (Chen et al., 2013). AKT promotes OC survival by regulating cytoskeletal fusion and migration. Conversely, knocking down the AKT gene decreases osteocalcin levels and bone mass (Ma et al., 2021).

GSK3β negatively regulates various signaling pathways and plays a crucial role in bone remodeling. Selective knockdown of GSK3β leads to premature growth plate phenotypes, resulting in shorter bones and developmental delays. Histological analysis also reveals an increase in OC and apoptosis in the skeletal tissues of GSK3β knockout mice (Bali et al., 2021). Research indicates that GSK3β may function as a kinase, promoting NFATc1 nuclear translocation through phosphorylation (Amirhosseini et al., 2018). However, other studies suggest that GSK3β negatively impacts OC differentiation, leaving its specific role in OC unclear (Jang et al., 2011). PPARγ, however, enhances the differentiation of mature OC. Its three isoforms promote nuclear aggregation and bone resorption activity in OC, contributing to OP development (Chan et al., 2007). Conversely, this conclusion conflicts with other research suggesting that PPARγ does not regulate OC either *in vivo* or *in vitro* (Zou et al., 2016). Thus, the exact mechanism by which GSK3β and PPARγ stimulate OC differentiation remains unclear and warrants further study. This study’s results show that NEO inhibits GSK3β and PPARγ phosphorylation at 30 min but not at 60 min.

Under RANKL stimulation, PLCγ binds to the IP3 receptor, releasing calcium ions from the endoplasmic reticulum. This increases intracellular Calcium levels and activates calmodulin expression, leading to NFATc1 dephosphorylation and nuclear translocation (Zeng et al., 2020). The influx of extracellular calcium ions is central to calcium signaling pathways, playing a key role in cellular physiology, biochemical functions, and signal transduction (Smyth et al., 2010). Calcium ions enter OB through voltage-gated ion channels, increasing intracellular concentration. This induces phosphorylation of CAMK4, CAMK2, and Calmodulin1/2/3 to activate intracellular calcium responses and ultimately trigger NFATc1 expression (Boonrungsiman et al., 2012). This study shows that after RANKL activates calcium channels, NEO can inhibit the phosphorylation of several channel proteins. This regulation impacts intra- and extracellular calcium levels, consequently affecting the formation of mature OC.

In this study, we conclude that NEO, even at low concentrations, inhibits the formation of mature OC and reduces the expression levels of genes and proteins specific to OC differentiation. Our findings reveal that NEO suppresses phosphorylation in the MAPK/NF-κB/PI3K-AKT signaling pathways and exerts inhibitory effects on the calcium signaling pathway as well as GSK3β and PPAR γ protein expression.

Nevertheless, our comprehension of the mechanisms by which NEO inhibits OC differentiation and alleviates bone loss remains rudimentary, and its precise molecular targets have yet to be elucidated. Several critical aspects warrant further investigation: Firstly, the bioavailability of NEO presents significant challenges, encompassing its oral absorption efficiency, *in vivo* distribution patterns, and metabolic profile; Secondly, the long-term safety profile of NEO administration necessitates comprehensive evaluation. Exploring potential synergistic effects and optimal combination strategies could yield more effective therapeutic regimens. These multifaceted aspects demand rigorous scientific scrutiny in future studies to fully elucidate NEO’s potential as a novel therapeutic agent for OP and to optimize its clinical application.

## Data Availability

The original contributions presented in the study are included in the article/Supplementary Material, further inquiries can be directed to the corresponding author.

## Abbreviations

BMMs, bone marrow macrophages; FBS, Fetal bovine serum; M-CSF, Macrophage-Stimulating Factor; BV/TV, bone volume/tissue volume; OP, osteoporosis; OB, osteoblast; OC, osteoclast; Specific Pathogen Free (SPF); Tb. N, trabecular number; TB. Sp, trabecular separation; CCK-8, Cell Counting Kit-8. TRAcP, The tartrate-resistant acid phosphatase; TB. Th, Trabecular thickness; DMSO, Dimethyl Sulfoxide; NEO, Neoandrographolide; BMSCs, bone marrow-derived mesenchymal stem cells.
