# Supplementary material for: Neoandrographolide inhibits mature osteoclast differentiation to alleviate bone loss and treat osteoporosis
Source: Front Pharmacol. 2025 Feb 11;16:1466057. doi: 10.3389/fphar.2025.1466057 (PMC11851123; doi:10.3389/fphar.2025.1466057)

CTSK:39 kDa

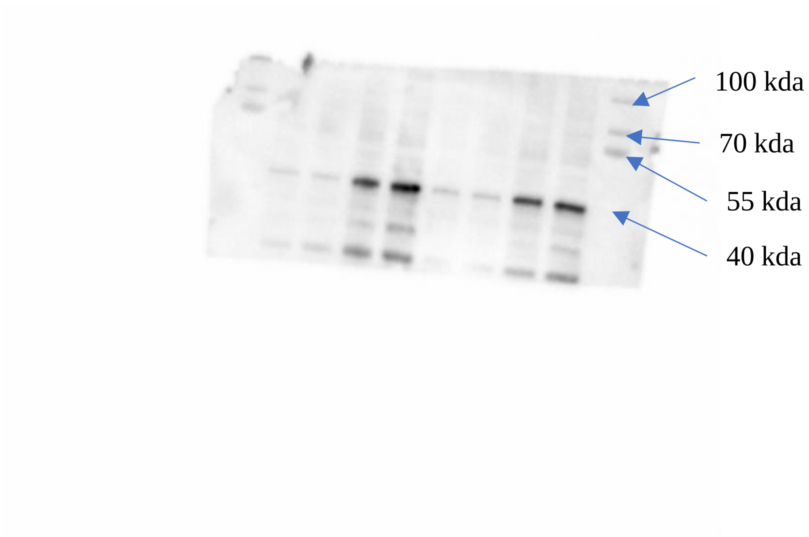

NFATC1:101 kDa

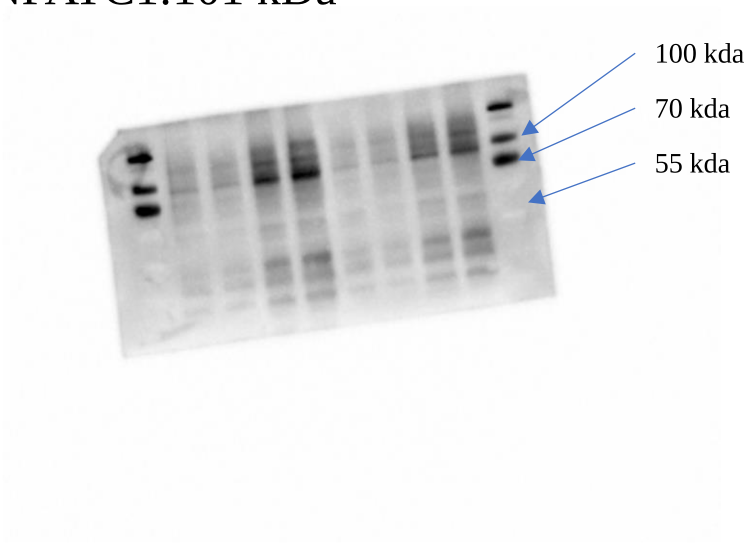

c-Fos:37 kDa

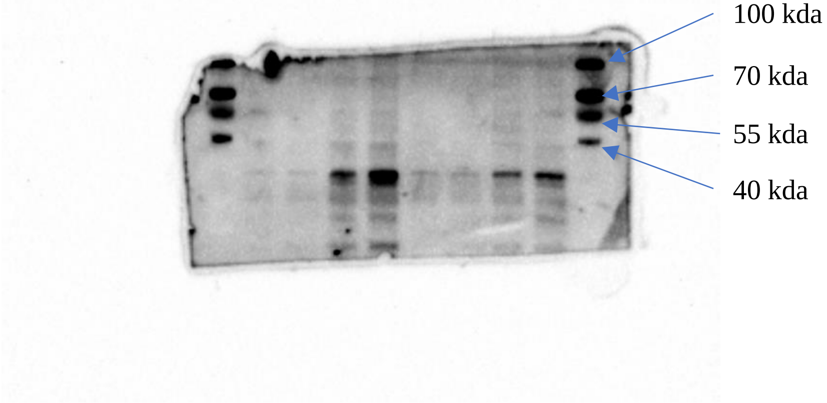

MMP9:105 kDa

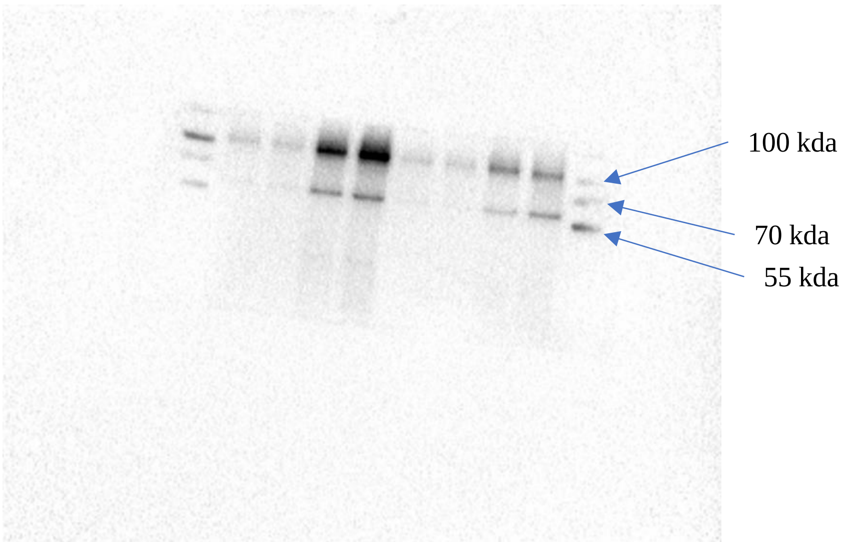

ERK/p- ERK :42/44 kDa

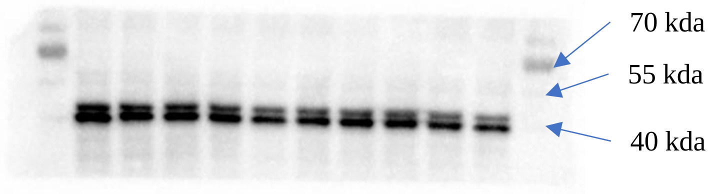

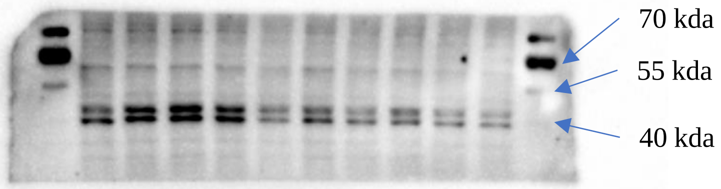

JNK/p- JNK :46/54 kDa

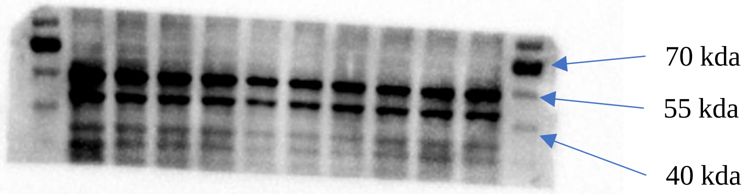

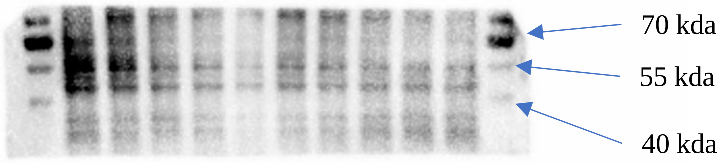

P38/p- P38 :41 kDa

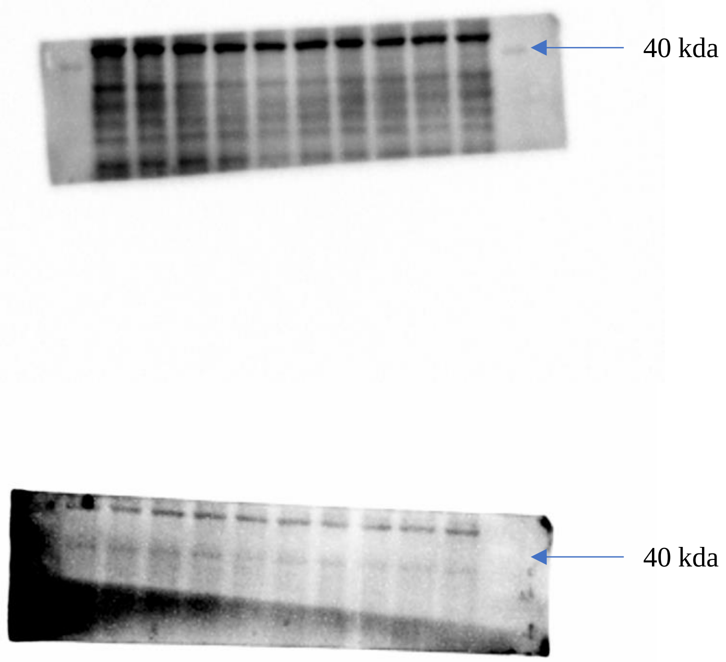

PI3K/p-PI3K:84 kDa

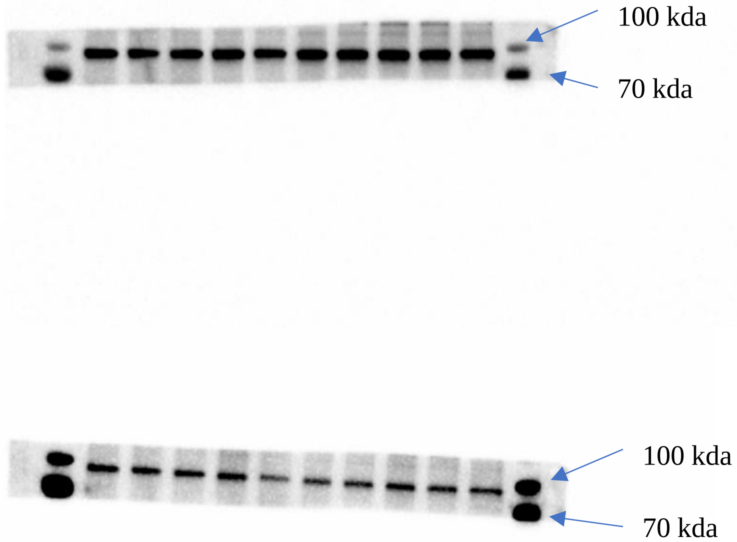

AKT/p- AKT :56 kDa

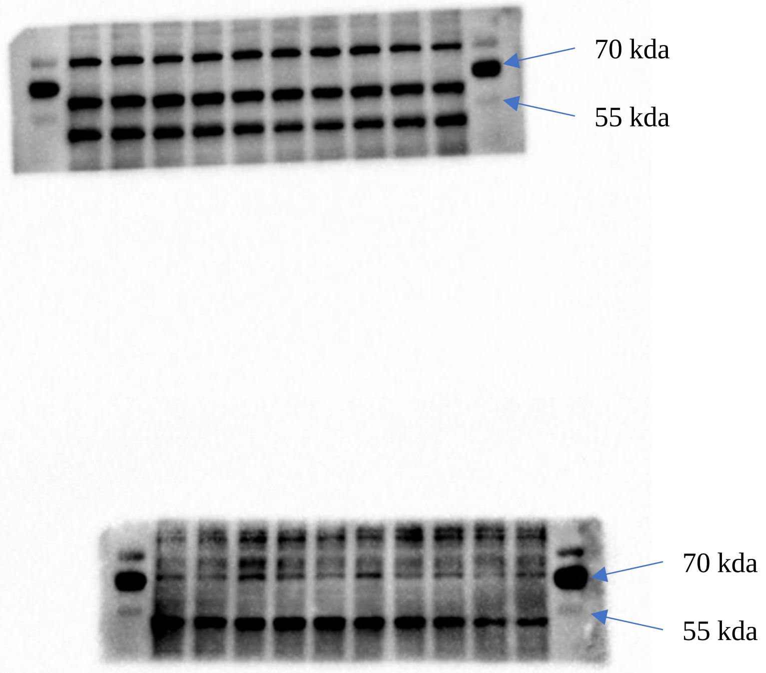

P65/p- P65 :65 kDa

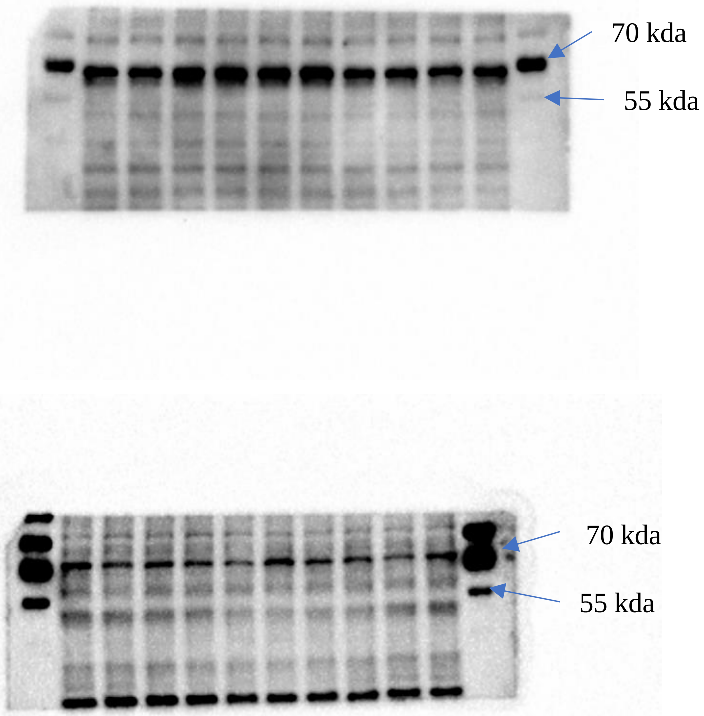

IκB-α:39 kDa

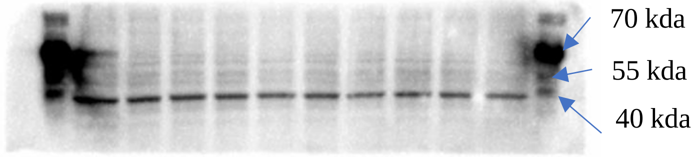

GSK3β/p-
GSK3β :48 kDa

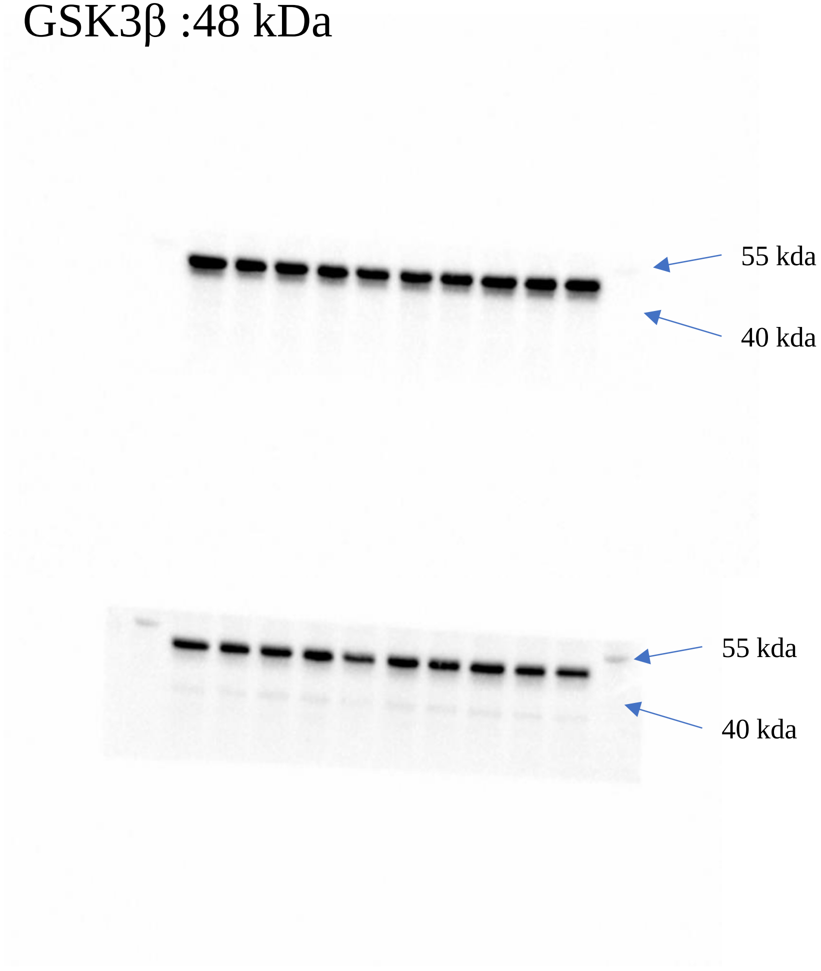

PPARγ/p-
PPARγ :57 kDa

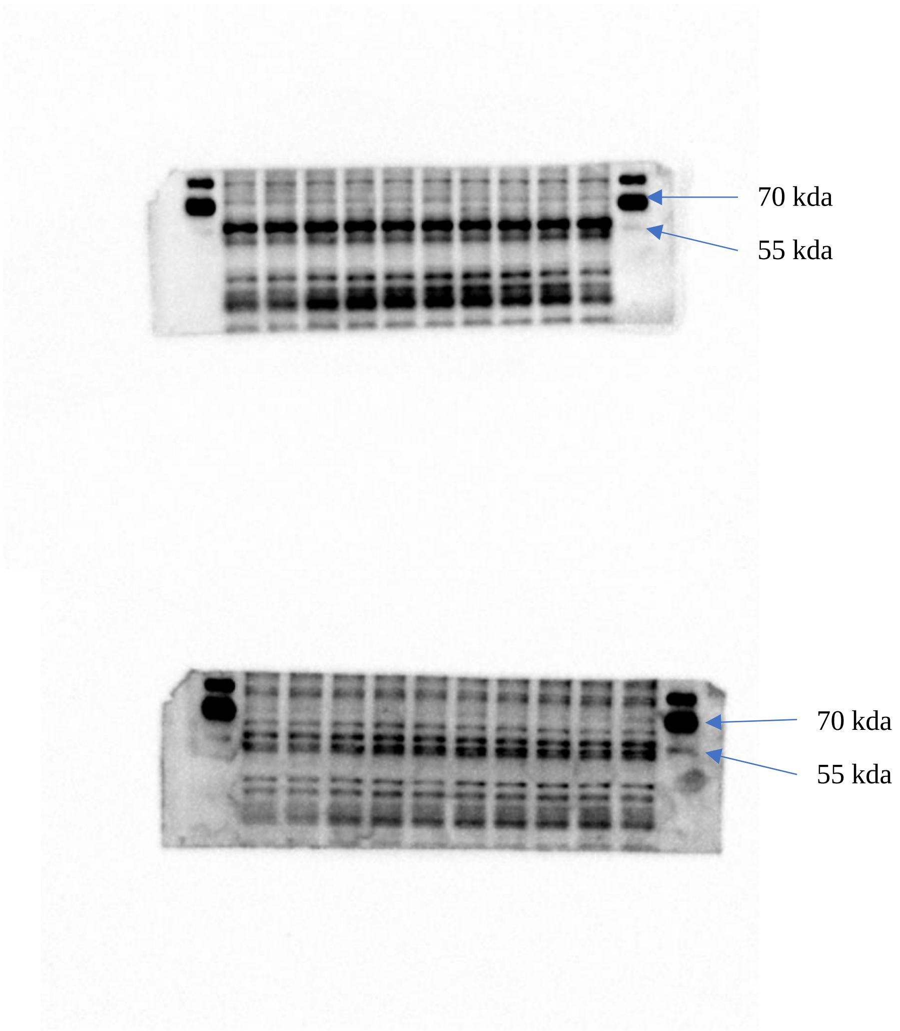

CAMK2/p- CAMK2 :50 kDa

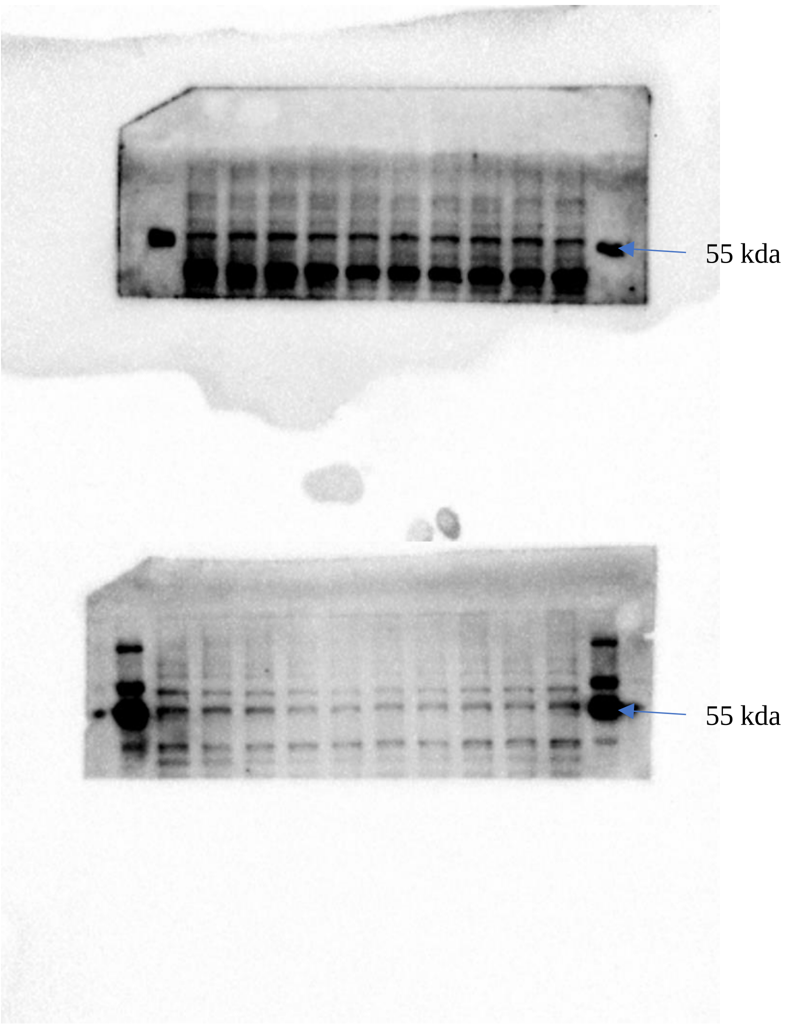

CAMK4/p- CAMK4 :55 kDa

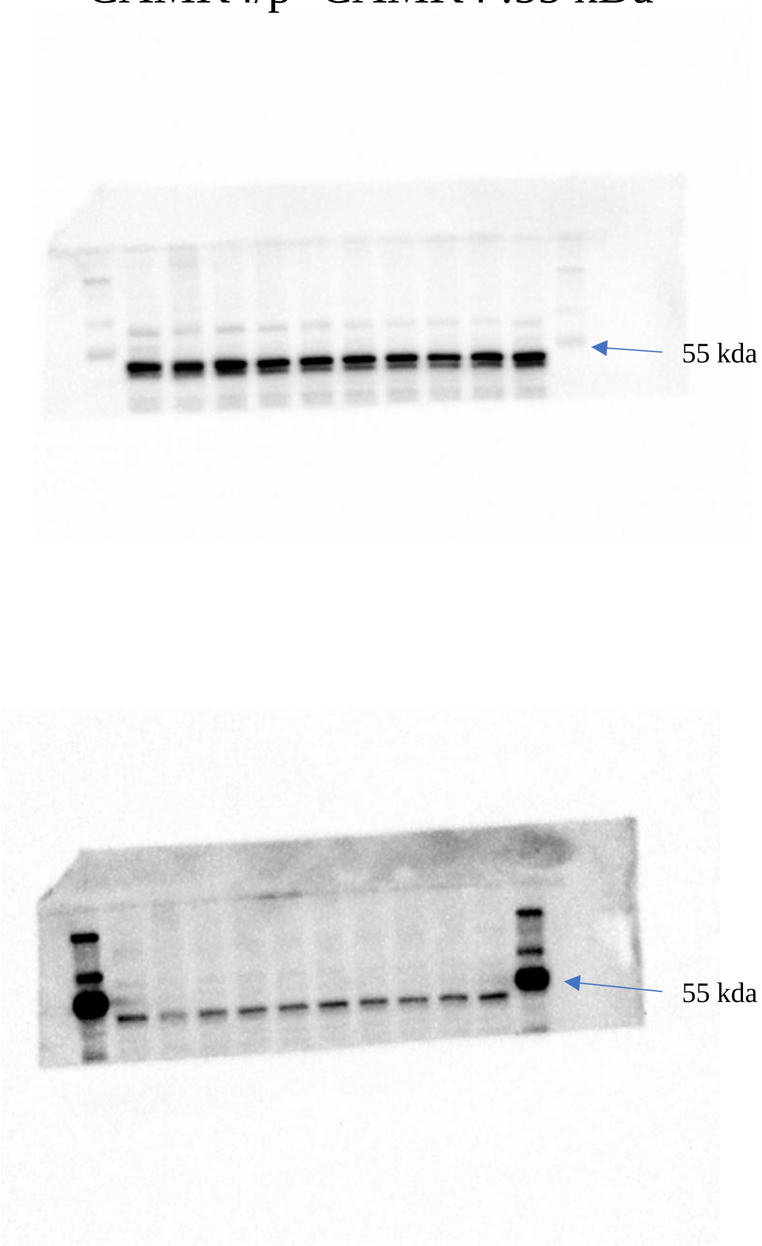

Supplement: Supplementary file 1 [file DataSheet1.pdf]
